# Vision transformer-equipped Convolutional Neural Networks for automated Alzheimer's disease diagnosis using 3D MRI scans

**DOI:** 10.3389/fneur.2024.1490829

**Published:** 2024-12-16

**Authors:** Zhen Zhao, Pauline Shan Qing Yeoh, Xiaowei Zuo, Joon Huang Chuah, Chee-Onn Chow, Xiang Wu, Khin Wee Lai

**Affiliations:** ^1^Department of Electrical Engineering, Faculty of Engineering, Universiti Malaya, Kuala Lumpur, Malaysia; ^2^Department of Biomedical Engineering, Faculty of Engineering, Universiti Malaya, Kuala Lumpur, Malaysia; ^3^Department of Psychiatry, The Affiliated Xuzhou Oriental Hospital of Xuzhou Medical University, Xuzhou, Jiangsu, China; ^4^School of Medical Information Engineering, Xuzhou Medical University, Xuzhou, Jiangsu, China

**Keywords:** Alzheimer's disease, classification, Convolutional Neural Network, magnetic resonance imaging, transformer

## Abstract

Alzheimer's disease (AD) is a neurodegenerative ailment that is becoming increasingly common, making it a major worldwide health concern. Effective care depends on an early and correct diagnosis, but traditional diagnostic techniques are frequently constrained by subjectivity and expensive costs. This study proposes a novel Vision Transformer-equipped Convolutional Neural Networks (VECNN) that uses three-dimensional magnetic resonance imaging to improve diagnosis accuracy. Utilizing the Alzheimer's Disease Neuroimaging Initiative (ADNI) dataset, which comprised 2,248 3D MRI images and diverse patient demographics, the proposed model achieved an accuracy of 92.14%, a precision of 86.84%, a sensitivity of 93.27%, and a specificity of 89.95% in distinguishing between AD, healthy controls (HC), and moderate cognitive impairment (MCI). The findings suggest that VECNN can be a valuable tool in clinical settings, providing a non-invasive, cost-effective, and objective diagnostic technique. This research opens the door for future advancements in early diagnosis and personalized therapy for Alzheimer's Disease.

## 1 Introduction

Alzheimer's disease is an increasingly severe neurodegenerative ailment that poses a significant worldwide health issue. Countless individuals and their families are impacted by the inexorable advancement of this condition, which deprives sufferers of their cognitive faculties and self-reliance. Characterized by memory loss, cognitive decline, and structural brain changes, AD poses a significant burden on healthcare systems worldwide. Alzheimer's disease, marked by amnesia, cognitive deterioration, and alterations in brain structure, imposes a substantial strain on global healthcare systems. A recent report estimates that there were 57.4 million dementia patients worldwide in 2019 and that figure is predicted to rise to ~152.8 million by 2050 (1).

Timely detection of AD is crucial. It enables prompt medical and therapeutic interventions and empowers patients and caregivers to strategize for the future. Studies have demonstrated that early therapies can decelerate the advancement of the disease, thereby enhancing the patients' quality of life (2). So, the pursuit of precise and timely diagnostic techniques remain a vital concern in the field of Alzheimer's research. Nevertheless, the task of diagnosing AD remains a daunting endeavor. Conventional diagnostic methods frequently depend on clinical evaluations, cognitive exams, and neuroimaging techniques. Although these procedures are helpful, they are prone to human error, may lack sensitivity in the initial phases of the disease and can be intrusive or expensive. Hence, there exists a demand for diagnostic techniques that are more precise, unbiased, and non-intrusive. Three-dimensional magnetic resonance imaging (3D MRI) scans are utilized in this context. MRI technology provides a non-invasive method to observe the structural changes in the brain, making it a suitable option for diagnosing AD (3). 3D MRI scans offer comprehensive and multi-dimensional insights into the structure of the brain, allowing for the identification of minor anomalies related to the disease.

Furthermore, recent breakthroughs in deep learning have completely transformed the field of medical image analysis (4). Deep learning algorithms, especially Convolutional Neural Networks (CNN), like VGG (5), ResNet (6), GoogLeNet (7), DenseNet (8), etc., and Vision Transformers (ViT) (9), which are inspired by neural networks, are highly proficient in extracting detailed patterns and features from complex medical images. Their capacity to autonomously acquire knowledge and adjust to data has unveiled novel horizons in the automation of disease diagnosis. Although deep learning and medical imaging have made tremendous strides, the diagnosis of AD using existing methods frequently struggles to appropriately identify complex spatial patterns in volumetric MRI data. The inadequacy of current models to efficiently include global context information may restrict their diagnostic accuracy, particularly during the early stages of the ailments. Due to this research gap, a novel model that improves feature extraction and discriminating abilities by utilizing the benefits of both pure 3D CNNs and mechanisms inspired by vision transformers are required.

In order to address this significant deficiency, this study aims to achieve two main research goals: firstly, to create a novel 3D CNN model with adaptations inspired by the vision Transformer capable of accurately distinguishing AD from other neurodegenerative disorders using 3D MRI scans, and secondly, to assess the effectiveness of the model on AD diagnosis. The foundation of this research is based on the notion that proposed pure 3D CNN model, enriched with vision transformer-inspired adaptations, will demonstrate improved diagnostic efficacy in comparison to traditional approaches. The suggested model has the potential to enable more precise differentiation among various stages of AD. The significance of this study includes that the accuracy of identifying AD, especially during its early phases, could be greatly improved by the suggested model. Another significant contribution of this research is the creative incorporation of vision transformer-inspired mechanisms into a pure 3D CNN architecture. This paper presents several innovative contributions to the field of Alzheimer's Disease diagnosis:

- Introduction of VECNN, integrating Vision Transformers and Convolutional Neural Networks.
- Proposal of a modified residual block distribution in ResNet-50, adopting a Swin Transformer-inspired ratio of 1:1:3:1.
- Implementation of non-overlapping convolutions with a 4 × 4 × 4 size and stride of 4 to reduce redundancy and computational complexity.
- Adoption of spatial separable convolution to enhance feature representation by effectively capturing spatial and channel relationships, thereby reducing computational demands.

## 2 Related work

Accurate and prompt identification of AD is crucial for the management and care of patients. Conventional methods for identifying AD, on the other hand, rely primarily on subjective and time-consuming clinical observation and behavioral evaluations. The diagnostic methods for AD have predominantly focused on clinical evaluations, cognitive exams, and two-dimensional MRI scans. The advancement of machine learning has led to the development of more effective and convenient computer-aided diagnostics that decrease costs while increasing accuracy.

Traditional machine learning methods, such as Support Vector Machines (SVM), Random Forest (RF), etc.. Develop a classifier that can accurately distinguish individuals with AD from individuals in other groups using features like cortical thickness and gray matter volumes. Given SVMs' capacity for effective performance and their transparent operational principles, they are widely utilized in various industrial and scientific fields. In a study by 10. Suk et al. (10), multi-kernel SVMs were employed for classifying integrated data derived from multi-modal inputs. Bi et al. (11) addressed the challenge of limited samples by introducing a novel clustering evolutionary random forest architecture. This approach was designed to handle multimodal data from ADNI, facilitating the detection of brain abnormalities and pathogenic genes. Suk and Shen (12), Suk et al. (10), and Suk et la. (13) introduced models that employ stacked Autoencoders (AEs) for feature extraction. The features extracted are then processed by a SVM for classification. Recent studies have shown that combining textural features reflecting local functional activity, such as the amplitude of low-frequency fluctuation, with structural MRI can improve diagnostic performance for Alzheimer's disease and amnestic mild cognitive impairment. These findings highlight the potential of multimodal radiomics techniques in enhancing early diagnosis accuracy (14).

The development of these trends has been further accelerated by the wide adoption of deep learning. Recent research has showcased the capacity of neural networks to retrieve complex patterns from medical images. The utilization of 3D MRI scans has become prominent in diagnosing AD due to its ability to provide more comprehensive structural information. Multiple research have investigated the possibility of using MRI to diagnose AD. Mainly, the methods consist of 2D sliced-based and 3D voxel-based approaches. The sliced-based method simplifies networks by avoiding the need to handle millions of parameters during training. Nevertheless, sliced-based method sacrifices the spatial relationships between neighboring picture slices. Voxel-based methods have the advantage of capturing the 3D information in brain scans, but they come with the drawback of intensive computing requirements and a large number of features. Jain et al. (15) used transfer learning with VGG16, pre-trained on ImageNet (16), for AD classification from 3D MRI slices converted to 2D. CNN was evaluated by Basaia et al. (17) on ADNI and a private dataset called Milan. The findings shown that CNN achieved a classification accuracy of 99% for distinguishing between AD and HC in the ADNI dataset, and 98% in the ADNI + Milan dataset., and 75% on both datasets for cMCI and sMCI detection. Abrol et al. (18) employed a 3D ResNet for AD diagnosis, achieving a prediction accuracy of 83% through domain transfer learning. Transfer learning was employed to adapt the trained model from MCI detection, which utilized 3D gray matter images as input, to the task of HC and AD classification. To assist with reducing the dimensionality of feature vectors, Alinsaif et al. (19) customized a CNN using the 3D shearlet transform and obtained a highest classification accuracy of 92.78%. Kruthika et al. (20) employed a two-stage classifier, comprising a Gaussian Naive Bayes Classifier, an SVM, and a KNN classifier, resulting in an accuracy rate of 90.47% when classifying HC/MCI/AD.

3D CNNs have demonstrated promise by learning spatial dependencies in neuroimaging tasks. Liu et al. (21) constructed a 3D DenseNet model to acquire information from the 3D patches that were extracted according to the classification task's hippocampus segmentation outcomes. Yeoh et al. (22) implemented 3DCNNs for the purpose of identifying knee osteoarthritis and employed transfer learning to enhance the model's performance. Shoaib et al. (23) utilized a convolutional neural network to automate the process of segmenting the left ventricle in order to analyze heart activity and diagnose cardiovascular illness. Korolev et al. (24) demonstrated that the residual and simple 3D CNN performed similarly in classifying AD against MCI and HC. Wang et al. (25) introduced a model where each classifier utilizes DenseNet as backbone, incorporating dense layers and activation functions. The 3D DenseNet is configured and trained separately, then a voting method is used to merge probabilistic results from these different classifiers. Cui et al. (26) proposed a CNN and RNN integrated classification framework that obtained 91.33% classification accuracy for AD and HC. For the purpose of diagnosing Alzheimer's illness, Lim et al. (27) trained a CNN from scratch, and its performance was compared to that of pretrained VGG and ResNet-50. Recent studies showcased the potential of ViT models in image classification tasks, raising questions about their applicability in medical image analysis. The self-attention (28) mechanisms of ViT facilitate the learning of global context, which could potentially be advantageous when conducting complex tasks like diagnosing AD. The integration of mechanisms inspired by vision transformers into CNN for neuroimaging may be able to maximize the benefits of both models by utilizing CNNs to capture local spatial patterns and vision transformer mechanisms to improve global contextual understanding. Zhao et al. (29) performed a series of experiments using 2D CNN and ViT, demonstrating the effectiveness of ViT on the task of AD diagnosis.

Despite substantial advancements, there continue to be obstacles in achieving a high level of diagnostic accuracy, specifically for the initial phases of AD. The requirement for interpretability and robustness in deep learning models for medical diagnosis continues to be a primary focus. Besides, generalization of the models is hindered by differences in picture resolution, data preprocessing, and acquisition methods of datasets for training and evaluating AD diagnosis models. Although there has been progress, there are still some unresolved questions in the current body of literature. It is worth mentioning that there is a lack of thorough investigation on the use of 3D VECNN models for diagnosing AD using MRI scans. The complete clarification of the possible benefits and constraints of this method is still pending, serving as the main motivation for this investigation. Research in this domain extends beyond prior studies that mainly concentrated on 2D MRI scans by incorporating MRI images and utilizing 3D VECNN models. The purpose of this methodological divergence is to utilize the detailed spatial information provided by 3D data and apply the capabilities of the ViT architecture for the examination of medical pictures. To summarize, the existing study highlights the increasing importance of using MRI-based diagnostics and deep learning techniques in the field of AD research. This research contributes to the current literature by investigating the capabilities of VECNN models when used with 3D MRI data, filling a significant gap in past studies.

## 3 Materials and methods

### 3.1 Dataset

The datasets utilized in this study is the Alzheimer's Disease Neuroimaging Initiative (ADNI). The ADNI dataset was accessed through the ADNI database (adni.loni.usc.edu). The 3D MRI scans were obtained utilizing scanners with varying magnetic field strengths, including 1.5T and 3T. The dataset included 188 AD, 401 MCI, and 229 NC subjects. This study utilized a total of 2,248 MRI scans obtained from 818 participants in the database. Specifically, only the standard 1.5T T1-weighted sMRI data were included in the analysis. The ADNI dataset encompasses a diverse range of participants, with age spanning from 55 to 90 years, representing both genders. The training, validation, and test sets are split according to a ratio of 8:1:1. In details, in the three way classification task, among the 2,284 samples, 1,827, 228, and 229 samples are divided into the training set, validation, and test set. A subject-level split strategy was adopted during the cross-validation process.

**Figure 1:**
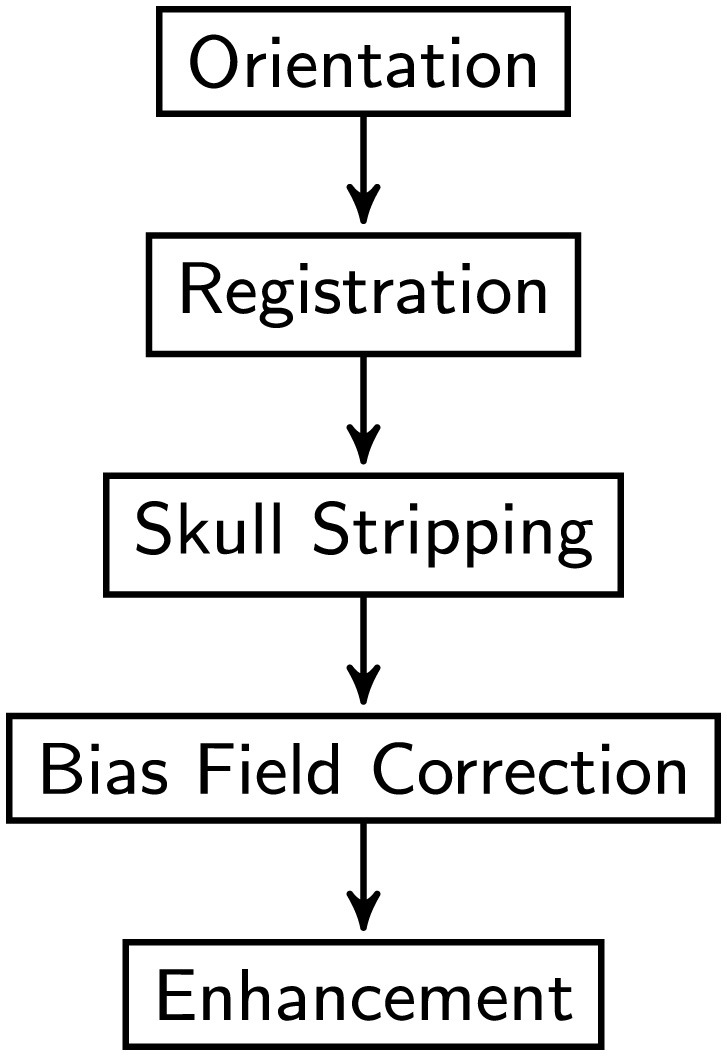
Image pre-processing procedure overview.

Prior to model training, all MRI scans underwent preprocessing procedures. As shown in Figure 1, the flowchart outlines the sequential steps involved in image preprocessing, Orientation, Spatial Registration, Skull Stripping, Bias Field Correction, Enhancement, and Normalization to a common anatomical template. Registration is a method to spatially align image scans to ensure the correspondence of anatomy across modalities, individuals, and studies. It registers multiple images of the same subject or distinct subjects into a common coordinate system. Skull-stripping or brain extraction means removing the non-brain tissues like skull, fat, eyes, etc., and remaining gray matter (GM), white matter (WM), Cerebrospinal fluid (CSF), etc. in the brain scan. Accurate removal of the non-brain tissues is crucial for obtaining valid results, as the presence of these tissues could introduce noise. In medical imaging, specifically MRI, the non-uniform intensity distribution in the image is referred to as the bias field or intensity inhomogeneity. The bias field can considerably affect the accuracy of the subsequent preprocessing process.

**Figure 2:**
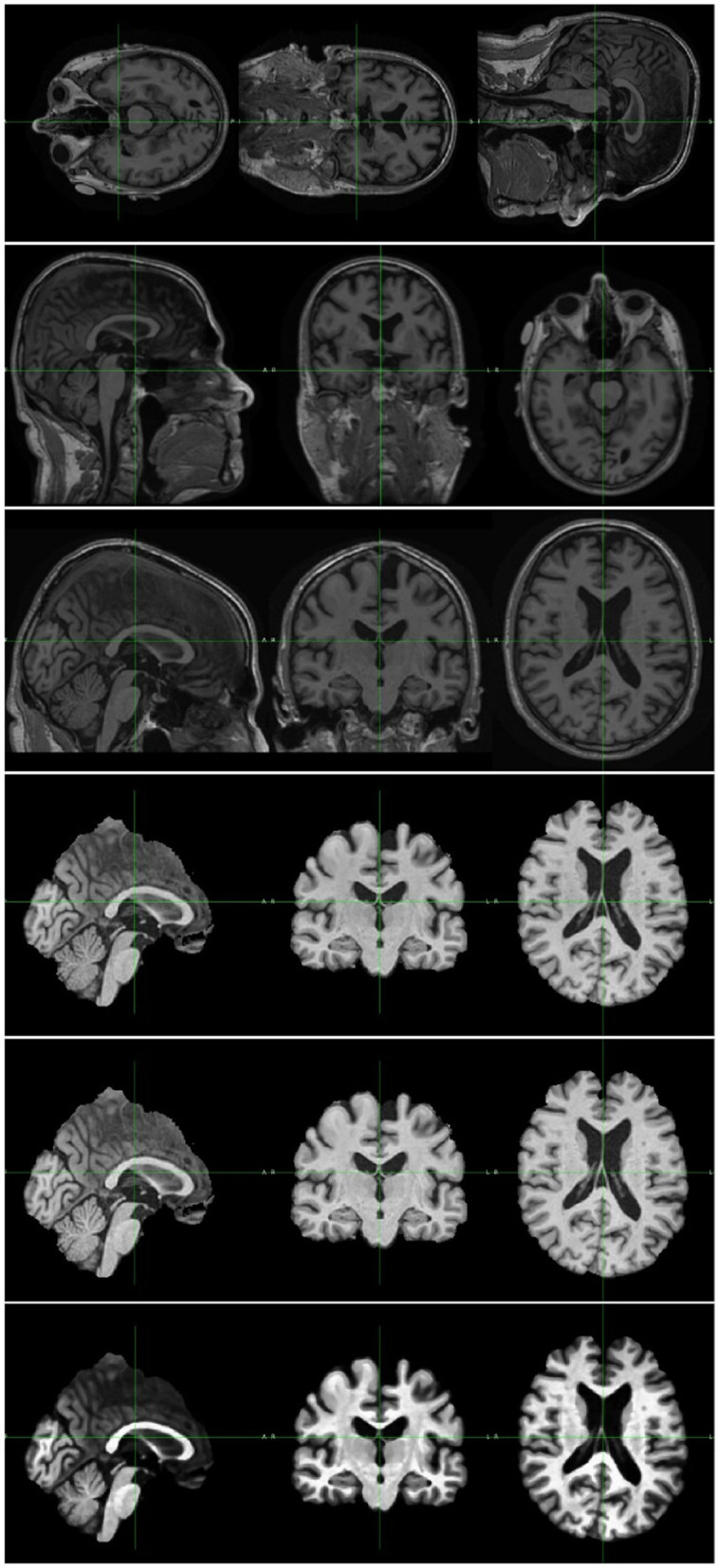
Image pre-processing sample. The top image is the original MRI scan. The rest are samples of image after each preprocessing step, including, orientation, registration, skull stripping, bias field correction, and enhancement.

First, the orientation tool in the FMRIB Software Library (FSL) (30) is used to match the image's orientation. Next, FSL's FLIRT (the FMRIB's Linear Image Registration Tool) is utilized for the purpose of image registration, which is essential for spatial correspondence. By employing FSL's Brain Extraction Tool (BET) (31), skull stripping ensures precise identification of brain tissue. The N4 method from ANTs (32) is used to provide bias field correction to address intensity gradients. Lastly, to increase visual quality and get the pictures ready for further analysis, image enhancement techniques such as median filtering, rescaling, and histogram equalization are applied. Quality control checks were performed to ensure data integrity. Data augmentation techniques were applied to the training subsets of dataset to enhance the diversity of the training samples. In this research, the data augmentation was implemented dynamically. As a result of the dynamic generation of each augmented image throughout the training process, the total amount of training data was significantly increased. In details, spatial augmentation techniques such as random flip, random affine, and random elastic deformation were employed with the possibility of one third. Intensity augmentation methods including random blur, random motion, random ghosting, and random noise were implemented with the possibility of one forth. Figure 2 depicts the raw image and the images generated after each preprocessing step. Addressing the uneven distribution of diagnostic categories, the data imbalance was mitigated by assigning weights to each class when calculating the loss to ensure balanced class representation. In summary, this study utilized the ADNI dataset, encompassing a wide range of subjects and clinical profiles. The integration of these datasets allowed us to conduct a comprehensive investigation into the diagnostic effectiveness of the proposed model in distinguishing different stages of AD.

### 3.2 Network architecture

This paper proposes a VECNN model architecture designed for automated AD diagnosis using 3D MRI scans. The primary objective of the proposed model is to leverage the spatial richness of 3D MRI data while using the power of the Vision Transformer architecture for extracting and classifying features with long-range dependencies. To integrate conventional CNN with ViT, two approaches can be employed: using a ViT as the backbone with integrated CNN characteristics or using a CNN as the backbone with incorporated ViT components. The latter approach is adopted in this study due to the high computational complexity associated with ViTs, particularly in 3D applications. Self-attention assesses relationships among all pairs of spatial positions within an image, allowing the model to concentrate on globally relevant regions. This suffers significant processing costs, particularly for high-dimensional datasets such as 3D MRI. Spatial separable convolution divides a full 3D convolution kernel into smaller, independent operations, such as horizontal and vertical convolutions. This decreases computing complexity while preserving the capacity to identify patterns in various spatial orientations. While less expressive than self-attention, spatial separable convolutions can mimic its functionality by facilitating information transfer over long spatial areas with reduced parameters and lower computation. When combined with suitable receptive field tuning and supplementary layers, they can attain a level of global feature integration similar to that of self-attention. This method enables efficient capture of local spatial patterns through CNNs while enhancing global contextual understanding with ViT-inspired mechanisms. The network is constructed using the 3D ResNet-50 architecture as its backbone, drawing inspiration from the enhancements introduced by ConvNeXt (33) to the ResNet framework. The ResNet-50 is regarded as the initial benchmark for commencing the training process in accordance with the training procedure of the vision transformer. A CNN typically comprises many convolutional layers, with each layer being subsequently followed by an activation function and batch normalization. The purpose of inserting max-pooling layers between convolutional layers is to extract hierarchical information and reduce spatial dimensionality. A global average pooling layer is frequently included in the CNN's last layer. The first part of a typical ViT network is patch embedding where the input image is patchified and embedded. Patchifying means to divide the input image into fixed-size patches. The patchified images are then linearly embedded into a flat vector format.

The initial design of the distribution of building blocks organized across blocks in ResNet was primarily based on empirical evidence [3, 4, 6, 3]. The conv4 layer as a substantial component was specifically designed to generate an feature map of 14 × 14 that is intended to be consistent with further tasks such as object detection. Swin-T adhered to the same approach, however with a slightly modified ratio of the number of stacked blocks, which was 1:1:3:1. The ratio for larger Swin transformers, including Swin-S, Swin-B, and Swin-L is [1, 1, 9, 1]. Following this design, the ratio of the number of stacked blocks in ResNet-50 was modified. In summary, the number of Residual Block in each level of the proposed model has been rearranged to correspond with the Swin Transformer's 1:1:3:1 ratio from 3:4:6:3. According to the distribution, the number of blocks that were present in each of the four layers was as follows: [3, 3, 9, 3]. This change slightly improved the accuracy.

The proposed model accepted 3D MRI scans as input data, represented as 3D tensors with dimensions [*C, H, W, D*], where *C* denotes the number of channels, *H* is the height, *W* is the width, and *D* is the depth. After preprocessing, the input size of the proposed model is (1, 112, 112, 112). Considering the considerable redundancy that comes with natural images, CNN and ViT employed comparable techniques to reduce the input to a proper size at the network's beginning. In earlier CNN designs, the initial layers often utilized larger kernels to capture broader spatial features. Vision Transformers, on the other hand, divided the input into a few patches. Given that ResNet was trained on datasets typically featuring larger images than 224 × 224, starting with a 7 × 7 convolutional layer might be too aggressive as a stem convolution for this task, especially considering the input size is 112 × 112 × 112.

Both CNN and Vision Transformers employ stem convolution to efficiently reduce the size of the input image to an appropriate feature map. The stem convolution in the vanilla ResNet architecture consists of a 7 × 7 convolutional operation with a stride of 2, which is then followed by a max pooling operation. It leads to a four times downsampling of the input. In the vision transformer, the stem convolution is intensified to a higher degree. The stem convolution in vision transformer is equivalent to employing a non-crossed convolution with dimensions of 14 × 14 or 16 × 16, or a fusion of a large convolution kernel and a large step size. The Swin Transformer also employs a comparable architecture but it uses a strategy similar to 4 × 4 disjoint convolutions for the initial downsampling. The proposed network architecture utilized non-overlapping convolutions with a size of 4 × 4 × 4 and a stride of 4. This change improved the accuracy by 0.29%. This demonstrates that the stem convolution of ResNet can be replaced with simple non-overlapping convolution, resulting in similar or even better performance.

Dilated convolution is another technique that could be used to obtain a wider receptive field. By using this technique, the receptive field is increased without using more parameters. In a standard convolution, each element of the kernel is applied to the input image or feature map without any gaps between them. In the case of dilated convolution, the convolutional kernel is expanded by introducing gaps between its components. The spacing between these elements is determined by the dilation rate. In this investigation, convolutions with a dilation factor of 2 were assessed. However, no discernible enhancement in performance was observed under these experimental conditions. Implementing the dilated convolution reduced the accuracy by 1.16%. Although dilated convolutions are effective at capturing long-range dependencies, the sparse sampling caused by dilatation may make it difficult for them to capture fine-grained local information. This limitation can be crucial in this task where fine details are essential.

The utilization of a larger convolutional kernel, such as a 7 × 7 kernel, conferred the advantage of an expanded receptive field. This extended receptive field contributed to enhanced modeling of long-range dependencies within the data. However, large kernels required more computations and consume more memory, leading to increased computational complexity. A golden standard proposed in VGG (5) is to use a stack of 3 × 3 × 3 kernel to replace 7 × 7 × 7 kernel. By the way, non-linearities between convolutional operations can be introduced by stacking numerous layers of small kernels.

This study also makes advantage of the spatial separable convolution design (34). Spatial separable convolution which used in Xception (34) and MobileNet (35) is the application of pointwise convolution after depthwise convolution. The weighted sum operation in self-attention is analogous to depthwise convolution. Channel and spatial mixing are separated by employing a combination of depthwise and pointwise convolutions. This approach ensures that the spatial and channel dimensions of the data are processed independently, without being mixed during the convolutional operation. Specifically, the pointwise convolution exclusively integrates information along the channel dimension, while the depthwise convolution solely combines data across the spatial dimensions. The spatial depthwise convolutional layer's placement has been shifted to precede the 1 × 1 × 1 convolutions, reflecting the design found in models like MobileNetV2 (36), EfficientNet (37), and EfficientNetV2 (38). In detail, the bottleneck structure from ResNet utilized a 1 × 1 × 1, a 3 × 3 × 3 and a 1 × 1 × 1 convolution. The bottleneck residual block from ResNet included a convolution that was 1 × 1 × 1, a convolution that was 3 × 3 × 3, and a convolution that was 1 × 1 × 1. Between every two convolutional operation, BN and Rectified Linear Unit(ReLU) are applied. The number of activation functions in the proposed model was reduced. In the Residual Blocks, we take the original scale and conduct a sequence of 3 × 3 × 3 convolution operations with BNs to expand the respective field, but we do not perform the ReLU activations that were previously performed. After the convolutions, Layer Normalization(LN) was implemented before further 1 × 1 × 1 convolutions. A minor change in employed in this proposed model was the activation function. ReLU is commonly employed in CNNs, including its usage in the architecture of the vanilla transformer. Many modern transformer architectures often employ the GELU (Gaussian Error Linear Unit) activation function as a substitute for the ReLU activation function. These structures comprise BERT (39) and GPT (40, 41).

**Figure 3:**
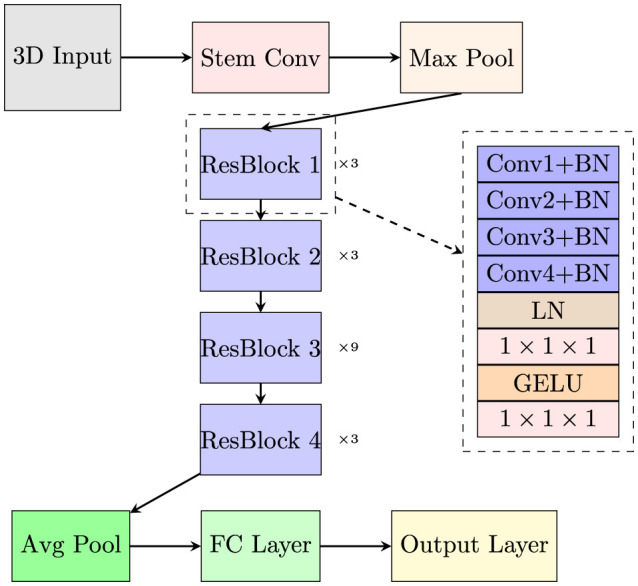
The overall network architecture of the proposed model.

The overall network architecture is depicted in Figure 3. In each layer, the initial Conv1 operation reduces the number of channels by half, whereas the subsequent convolutions decrease it by a factor of 4. The Conv4 introduces a stride, resulting in a halving of the spatial dimensions. Batch Normalization(BN) comes after every convolution. After the convolutions are complete, LN is applied. The activation function used within the ResBlock is GELU instead of ReLU to provide a smoother gradient for better model training. Following this, the 1 × 1 × 1 convolutions are used to increase the number of channels by a factor of 4. In summary, after each layer, the channel count of the features doubles, and the spatial dimensions are halved.

The proposed model is trained using Binary Cross-Entropy Loss and Cross-Entropy Loss according to the type of task and optimized with the AdamW optimizer. The study used an exponential learning rate scheduler with a multiplicative component for the decay of the learning rate, featuring an initial learning rate set at 5*e*−5. In this study, 10-fold cross-validation was employed to evaluate the performance of the proposed model. Key hyperparameters include a batch size of 16, and 150 epochs for training. The model is implemented using PyTorch (v3.9.7) and trained on a Ubuntu 20.04.3 LTS system equipped with an Intel Core i5 16-core 3.69 GHz CPU and a 12 GB NVIDIA GeForce RTX 3080Ti GPU. Every stage of the workflow for processing MRIs uses the FMRIB Software Library v6.0.

## 4 Result

To evaluate the model's diagnostic performance, the subsequent quantitative metrics are introduced, as defined by the following Equations 1–4:


(1) $$\begin{array}{l} Accuracy=\frac{TP+TN}{TP+TN+FP+FN} \end{array}$$

(2) $$\begin{array}{l} Precision=\frac{TP}{TP+FP} \end{array}$$

(3) $$\begin{array}{l} Sensitivity=\frac{TP}{TP+FN} \end{array}$$

(4) $$\begin{array}{l} Specificity=\frac{TN}{TN+FP} \end{array}$$

Here, *TP*, *TN*, *FP*, and *FN* represent *true positive*, *true negative*, *false positive*, and *false negative*, respectively.

The proposed VECNN model achieved an average accuracy of 92.14% for HC, MCI, and AD classification on the ADNI datasets, demonstrating its proficiency in AD diagnosis. In this 3-way classification task, the average sensitivity, specificity and precision values were 93.27, 89.95, and 86.84% respectively. The accuracy of the model for the three classes were as follows: 91.27% for HC, 89.52% for MCI, and 95.63% for AD. These results reflected the model's ability to differentiate between the different stages of Alzheimer's disease. The model exhibited an accuracy of 91.27% for HC. The sensitivity of 82.89% demonstrated a robust capacity to accurately identify HC samples, but it was slightly less than the specificity of 95.42%. This indicated that although the model effectively identified healthy controls, it could have resulted in some false negatives. A precision of 90% indicated that the majority of predicted HC samples were accurately identified as HC, hence validating the model's reliability for this category. The sensitivity for MCI was notably high at 94.29%, indicating the model's efficacy in identifying MCI cases. The specificity was 85.48%, suggesting that the model was more susceptible to interpreting non-MCI participants as MCI relative to other categories. The precision rate was 84.62%, indicating that the model's predictions of MCI were generally correct, though there was potential for enhancement in minimizing false positives. For AD, the model attained a specificity of 98.90%, indicating its efficacy in identifying non-Alzheimer's disease participants. The sensitivity of 83.33% was inferior to the specificity, suggesting that although the model effectively identified AD patients, it still produced some false negatives. The high precision of 95.24% indicated that most projected AD cases were accurate.

**Figure 4:**
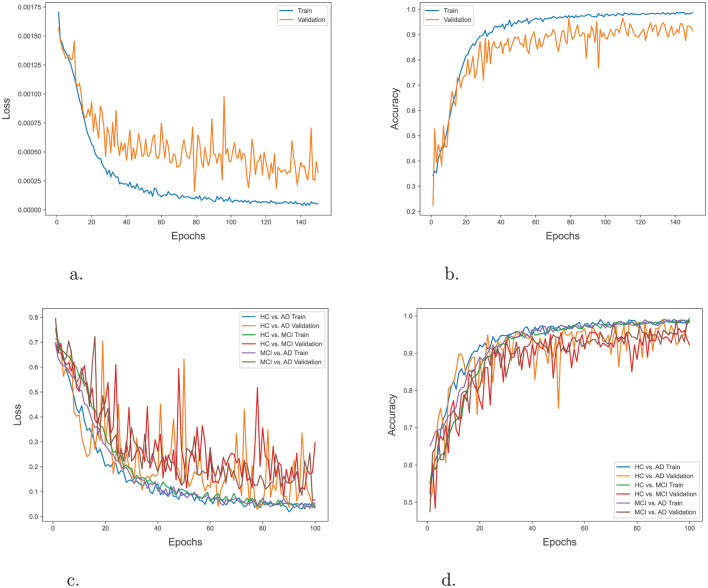
**(A)** Training and validation loss over epochs in 3-way classification. **(B)** Training and validation accuracy over epochs in 3-way classification. **(C)** Training and validation loss over epochs in binary classification. **(D)** Training and validation accuracy over epochs in binary classification.

In order to evaluate the training progress of our proposed model, the loss and accuracy of the model on both the training and validation datasets during multiple epochs in both 3-way and binary classification were tracked. Figures 4A, B illustrate the training progress and the performance of the model over epochs in the 3-way classification task. The training accuracy exhibited a consistent upward trend, suggesting that the model was efficiently learning from the training data. The validation accuracy shown improved gradually over time, eventually stabilizing at a threshold. This indicates that the model had achieved the ability to generalize effectively to unfamiliar data. Similarly, Figures 4C, D depict the advancement of training and the model's performance across epochs in the context of a binary classification task. The trends in these plots also suggest effective learning and strong ability to apply that knowledge to new situations.

A further evaluation is performed to validate the effectiveness of the proposed VECNN model in diagnosing AD by conducting a comparative analysis with several 3D models established in the literature. Multiple classification tasks are compared, such as HC/MCI/AD, HC/AD, HC/MCI, and MCI/AD. Table 1 presents the performance metrics, specifically accuracy, precision, sensitivity, and specificity, for each model and task.

**Table 1:** VECNN performance metrics comparative analysis (in %).

**Model**	**Task**	**ACC**	**PRE**	**SEN**	**SPE**
Kruthika et al. (20)	HC/MCI/AD	90.47	80.26	88.62	90.15
Cui and Liu (26)	HC/AD	91.33	–	–	–
Basaiaet al. (17)	HC/AD	98.20	–	98.10	98.30
Abrol et al. (18)	HC/AD	83.01	–	76.00	87.00
Liu et al. (21)	HC/AD	88.90	–	86.60	90.80
	HC/MCI	76.20	-	79.50	69.80
Alinsaif et al. (19)	HC/AD	94.44	–	95.65	92.31
Agarwal et al. (42)	MCI/AD	93.10	86.20	–	–
	HC/MCI/AD	87.38	86.38	87.51	–
Proposed	HC/MCI/AD	92.14	86.84	93.27	89.95
	HC/AD	94.07	93.06	95.65	97.10
	HC/MCI	91.21	85.71	94.12	88.52
	MCI/AD	91.19	84.62	93.33	80.49

Symbol “–” denotes no data.

As shown in Table 1, in comparison to previous studies, the VECNN exhibited superior diagnostic accuracy. When compared to existing models in the literature, the proposed model consistently outperforms them. As an instance, the 3D CNN developed by Basaia et al. (17) attained an accuracy of 98.20% for the HC/AD task on the ADNI and their own private dataset. The study conducted by Abrol et al. (18) demonstrates that their model achieves an accuracy of 83.01%, a sensitivity of 76.00%, and a specificity of 87.00% for the HC/AD task. However, these results indicate that their model performs less effectively compared to the proposed model. The study conducted by Alinsaif et al. (19) demonstrated a high accuracy rate of 92.78% for the task of distinguishing between HC and those with AD. In comparison, Liu et al. (21) reported that the accuracy for the HC/AD task was 88.90%, with a sensitivity of 86.60% and a specificity of 90.80%. For the HC/MCI job, the accuracy was 76.20%, with a sensitivity of 79.50% and a specificity of 69.80%. Kruthika et al. (20) present a model that implemented a 2-stage classification approach. In the first stage, a Gaussian Naive Bayes Classifier was used to determine whether an object belongs to the AD, MCI, or NC class, or if it is uncertain and should be rejected. In the second stage, SVM and KNN classifiers were applied to classify the objects based on the predictions from the first stage. Thier model achieves an accuracy of 90.47%, a precision of 80.26%, a sensitivity of 88.62%, and a specificity of 90.15% for the HC/MCI/AD task. In contrast, Cui and Liu (26) combined CNN and RNN for longitudinal analysis of structural MR images. The CNN captured spatial features, while the RNN extracted longitudinal features. Their result showed a higher accuracy of 91.33% for distinguishing between HC and individuals with AD. These models do not have the complete accuracy, sensitivity, and specificity metrics, which makes it difficult to directly compare them. Lastly, the model proposed by Agarwal et al. (42) utilized the 3D EfficientNet-b0 CNN to accurately categorize different stages of Alzheimer's disease. The EfficientNet-b0 model achieved an accuracy of 93.10% and a precision of 86.20% in the MCI/AD task. For the HC/MCI/AD task, the accuracy was 87.38%, the precision was 86.38%, and the sensitivity was 87.51%.

The findings suggest that the VECNN model exhibits potential as a precise and interpretable tool for diagnosing AD through the analysis of 3D MRI scans. This improvement underscores the potential of the VECNN architecture in the context of 3D MRI-based AD diagnosis. The model's proficiency in capturing spatial relationships within brain regions affected by the disease contributes to its enhanced diagnostic capability. Additionally, the observed performance improvement relative to 2D-based models implies potential for broader clinical relevance. To sum up, this study provides strong evidence for the efficacy of the VECNN model in automated AD detection utilizing 3D MRI data This model's outstanding quantitative performance makes it a viable tool for accurate and timely diagnosis, opening new avenues for AD research and clinical applications.

## 5 Discussion

The application of non-overlapping stem convolution, change for computation distribution, and spatial separable convolution are the primary characteristics of the proposed model. When compared to text, image data, particularly 3D medical imaging, contains a comparatively substantial amount of redundant information. In the process of feature extraction, non-overlapping convolution is helpful in reducing redundancy. Because the convolution operations do not overlap, each one processes a distinct and unique chunk of the input data. This can result in feature maps that are more efficient and contain fewer instances of redundant information. Besides, using non-overlapping convolutions can decrease the amount of computational work required. Due to the absence of overlap between the patches, the total number of convolution operations required is reduced compared to convolutions that involve overlapping. This can lead to accelerated calculations and reduced memory consumption. Using non-overlapping convolutions can simplify the structure of the network. By utilizing separate patches, the need to handle overlapping regions and their corresponding computations is eliminated, resulting in a simpler and perhaps more interpretable model. Adopting the Swin Transformer's approach to block distribution aligns the model with a proven design that effectively handles complex tasks. The Swin Transformer's block distribution has been shown to work well for various vision tasks, suggesting that similar benefits can be obtained by applying this strategy to the VECNN model. Extensive research has been conducted on the distribution of computing, as evidenced by studies (43, 44). The rearrangement of residual blocks to match the Swin Transformer's 1:1:3:1 ratio ensures that more computational power is devoted to the middle layers, which are crucial for capturing detailed and abstract features. The increased number of blocks in the middle layers allows the model to learn more hierarchical and abstract features. This is particularly important for tasks like AD diagnosis, where subtle differences in MRI scans need to be captured and understood at multiple levels of abstraction. At last, The concept of depthwise convolution in spatial separable convolution is similar to the weighted sum process in self-attention mechanisms employed in transformers. The shared characteristics between spatial separable convolution and transformer-based architectures allows for an effective combination of the two, resulting in an enhanced ability to capture both spatial and channel-wise dependencies. Spatial separable convolution effectively decreases the number of parameters and computational cost in comparison to traditional convolution by segregating spatial and channel mixing. This enhances the efficiency and accelerates the training and inference process of the model. The findings shed light on the possibility of deep learning models in addressing the pressing challenge of early and accurate diagnosis.

However, It is crucial to point out that the proposed VECNN model outperformed traditional CNN-based approaches, marking a significant advancement in the field. This performance gain may be attributed to the VECNN's ability to capture both spatial and contextual information within 3D MRI scans. The practical implications of the findings are substantial. Accurate and early diagnosis of AD is crucial for timely intervention and patient care. The VECNN offers a promising tool for clinicians, potentially reducing diagnostic errors and enabling early intervention strategies. Furthermore, it can aid in patient stratification for clinical trials and treatment planning. It is critical to recognize the study's limitations. First, even though the VECNN model performs well in terms of diagnosis, more extensive and varied external validation is necessary. Secondly, the interpretability insights might not fully represent the variety of model behavior because they are based on a limited quantity of cases. In order to improve diagnostic accuracy even more, future research directions include investigating new data modalities like voice, positron emission tomography (PET), and functional magnetic resonance imaging (fMRI). Furthermore, studies examining the robustness of the model across various scanner types and patient populations are crucial for practical translation. In conclusion, the study highlights the promising role of the VECNN in automating AD diagnosis from 3D MRI scans. While further research and validation are required, the findings pave the way for advancements in early diagnosis and personalized treatment strategies for this debilitating neurodegenerative disease. The authors anticipate that ongoing research in this field will not only refine diagnostic tools but also bring us closer to the ultimate goal of developing effective therapies for Alzheimer's disease, significantly improving the quality of life for affected individuals and their families.

## 6 Conclusions

This paper aimed to evaluate the effectiveness of the VECNN in this crucial healthcare domain. This study contributes to the increasing body of data that demonstrates the effectiveness of deep learning models in correctly identifying cases of AD in its early stages, which is crucial for intervention and therapy. The findings of this research have both practical and theoretical implications. In practice, the VECNN can be included in clinical workflows to improve early intervention facilitation, lower false negative rates, and improve diagnostic accuracy. This research contributes to the theoretical knowledge of CNNs and ViTs working together to analyze complicated 3D medical imaging data. Finally, several directions for further study are proposed. To begin with, external validation on larger and more varied datasets is necessary to verify the robustness of ViT-CNN. In addition, investigating the incorporation of supplementary data modalities, including functional magnetic resonance imaging and genetic data, may augment the accuracy of the diagnosis. To sum up, this research provides strong evidence that the VECNN can be used to automatically diagnose AD using 3D MRI data. It contributes to the development of AI-driven solutions in neurology and medical imaging, as well as better outcomes for those with AD.

## Data Availability

The original contributions presented in the study are included in the article/supplementary material, further inquiries can be directed to the corresponding authors.
